# Trust the Machine or Trust Yourself: How AI Usage Reshapes Employee Self-Efficacy and Willingness to Take Risks

**DOI:** 10.3390/bs15081046

**Published:** 2025-08-01

**Authors:** Zhiyong Han, Guoqing Song, Yanlong Zhang, Bo Li

**Affiliations:** School of Business Administration, Anhui University of Finance and Economics, 962, Caoshan Road, Bengbu 233030, China; hzyong@aufe.edu.cn (Z.H.); 3202300385@aufe.edu.cn (G.S.); 3202400113@aufe.edu.cn (Y.Z.)

**Keywords:** AI usage, self-efficacy, willingness to take risks, learning goal orientation

## Abstract

As artificial intelligence (AI) technology becomes increasingly widespread in organizations, its impact on individual employees’ psychology and behavior has garnered growing attention. Existing research primarily focuses on AI’s effects on organizational performance and job design, with limited exploration of its mechanisms influencing individual employees, particularly in the critical area of risk-taking behavior, which is essential to organizational innovation. This research develops a moderated mediation model grounded in social cognitive theory (SCT) to explore how AI usage affects the willingness to take risks. A three-wave longitudinal study collected and statistically analyzed data from 442 participants. The findings reveal that (1) AI usage significantly enhances employees’ willingness to take risks; (2) self-efficacy serves as a partial mediator in the connection between AI usage and the willingness to take risks; and (3) learning goal orientation moderates both the relationship between AI usage and self-efficacy, as well as the mediating effect. This research enhances our understanding of AI’s impact on organizational behavior and provides valuable insights for human resource management in the AI era.

## 1. Introduction

The rapid development of AI technology has resulted in its broad adoption in organizations. A recent global survey conducted by McKinsey in early 2024 reveals that 72% of enterprises have integrated AI technologies into their operations, positioning AI as a pivotal driver of digital transformation (McKinsey & Company, 2024). As a novel work tool, AI not only reshapes individual work practices but also fundamentally alters employees’ cognitive processes and behavioral patterns (Raisch & Krakowski, 2021). The advent of large language models, such as ChatGPT, has markedly intensified employees’ reliance on AI (Hu et al., 2023). However, the deep-level mechanisms through which AI usage affects employees’ psychological cognition and decision-making behaviors remain insufficiently explored, presenting important theoretical and practical challenges for understanding employee behavior in the AI era.

Existing research indicates that the widespread application of AI within organizations may influence individuals’ cognitive processes and behavioral patterns (Z. Zhang & He, 2024). However, there is a need for more systematic exploration regarding the underlying mechanisms involved. Current studies primarily investigate how AI influences organizational performance (e.g., Tang et al., 2022) and work design (e.g., Jia et al., 2024), while relatively few studies explore how AI usage influences the psychological and behavioral dimensions of employees. In particular, our understanding of how AI usage affects risk decision-making—an essential behavior for fostering organizational innovation—remains limited. Tang et al. (2022) define AI usage as employees engaging with various forms of AI to perform relevant tasks, including analysis, computation, and decision-making. Furthermore, AI usage may influence individuals’ self-perception and decision-making autonomy (Ahmad et al., 2023), subsequently impacting their willingness to take risks. Nonetheless, this potential mechanism has yet to receive a thorough theoretical explanation or empirical examination. Therefore, investigating how AI usage reshapes individual willingness to take risks through influencing psychological cognition holds significant theoretical and practical importance.

Social cognitive theory (SCT) provides an essential theoretical framework for understanding individual behavior within the context of artificial intelligence (AI). The theory asserts that individual behavior arises from the continuous interaction of individual characteristics (such as cognition and emotion), situational factors, and behavioral factors (Bandura, 1986). The widespread implementation of AI technology not only transforms environmental factors, such as organizational behavior, work design, and workflows (Jia et al., 2024; Tang et al., 2022) but also generates complex influences on individuals. For example, research by Tang et al. (2023) indicates that reliance on intelligent machines in the workplace can facilitate progress toward work goals and can subsequently enhance performance. However, this dependence may also pose risks to employee self-esteem and may lead to a decreased performance. This dual-impact mechanism suggests that AI usage, as an emerging environmental factor, necessitates a more in-depth psychological analysis to fully understand its effects on individual behavior. Within the Social Cognitive Theory framework, this triadic interaction provides a theoretical foundation for understanding individual willingness to take risks in AI contexts.

Within the framework of SCT, self-efficacy emphasizes an individual’s belief in their capability to successfully execute a task or accomplish a goal (Bandura, 1997). Such beliefs not only influence behavioral choices but also impact an individual’s persistence and emotional responses when confronted with challenges. In relation to artificial intelligence (AI), AI usage, as an emerging environmental factor, may influence individuals’ self-efficacy (personal factors), subsequently affecting their willingness to take risks. Specifically, as generative AI tools, including ChatGPT, become increasingly utilized, AI usage may impact individuals’ autonomy in learning, thereby moderating their self-efficacy (S. Zhang et al., 2024). As a critical determinant of behavior, an increase in self-efficacy may lead individuals to exhibit greater risk aversion (Krueger & Dickson, 1994). Furthermore, SCT underscores the importance of individual traits in shaping behavioral choices. VandeWalle et al. (2001) assert that an essential individual characteristic, or learning goal orientation, affects how individuals evaluate their external environment and how they respond emotionally. In AI contexts, those who possess a high learning goal orientation are inclined to perceive AI as a tool for development rather than a complete substitute, thereby enhancing the beneficial outcomes of AI usage on self-efficacy. Nonetheless, this moderating mechanism has yet to be systematically validated in the existing literature.

The primary objective of this research is to systematically investigate how AI usage reshapes employees’ willingness to take risks by affecting their self-efficacy, and to identify the boundary conditions of learning goal orientation in this process. Founded on social cognitive theory, this research develops a moderated mediation model to systematically address three key research questions: (1) how AI usage influences employees’ willingness to take risks; (2) whether self-efficacy mediates the relationship between AI usage and willingness to take risks; and (3) how learning goal orientation moderates the relationship between AI usage and self-efficacy, and subsequently influences the entire mediation process. This research makes three key theoretical contributions: First, it extends social cognitive theory into the AI context, revealing the pathways through which AI usage affects individual psychology and behavior, thereby enriching the theoretical understanding of AI impacts within the domain of organizational behavior. Second, this research introduces self-efficacy as a mediating mechanism, illuminating how AI usage affects the willingness to take risks and advancing insights into the psychological implications of technology usage. Third, analyzing how learning goal orientation moderates the relationship between AI usage and self-efficacy highlights essential personal traits that can foster the positive impacts of AI, offering valuable implications for management strategies in organizations. Through this integrated model, this study provides a novel theoretical perspective for understanding individual behavior formation mechanisms in the AI era and offers theoretical guidance for organizational management practice.

The subsequent sections of this study are organized as follows: The second section reviews relevant literature and proposes research hypotheses. The third section introduces the research methodology, including sample selection, variable measurement, and data collection procedures. The fourth section reports the empirical analysis results, including descriptive statistics, correlation analysis, and hypothesis testing. The fifth section discusses the conclusions of research findings, theoretical contributions, and practical implications. The sixth section discusses limitations and proposes future research directions.

## 2. Theoretical Foundation and Research Hypotheses

### 2.1. AI Usage and Willingness to Take Risks

AI, as a powerful transformative force, has deeply influenced multiple sectors of society and is considered to be central to the “Fourth Industrial Revolution.” As artificial intelligence technology becomes widely applied in organizational environments, the interactions between employees and AI systems have significantly influenced their behavioral decision-making (Z. Zhang & He, 2024). For instance, Shrestha et al. (2019) explored how organizational decision-making structures have changed with the rise in AI, noting that AI algorithms assist decision-makers in achieving faster, more accurate, repeatable, and low-cost decisions by extracting patterns and predictions from large datasets. Bag et al. (2021) investigated how AI influences decision-making processes within B2B markets. Studies indicate that AI can enhance decision-making capabilities by harnessing knowledge from customers, users, and the broader market. This research proposes that AI usage may encourage employees to take more risks by enhancing their information acquisition, analytical abilities, and predictive precision. According to Dewett (2006), the willingness to take risks is characterized by the tendency to accept certain job-related risks for the sake of achieving positive outcomes at work. Bandura’s (1986) social cognitive theory acts as the foundation for exploring how the use of AI impacts risk-taking willingness. This theory emphasizes that individual behavior is affected by a combination of environmental factors, cognitive processes, and observational learning. When employees use AI to assist in decision-making, the data analysis and predictive capabilities provided by the system may reduce uncertainty, thereby increasing their confidence in taking risks.

Empirical research indicates that the application of technological tools can improve decision-making processes and enhance decision quality, thereby reducing risks and promoting human–machine collaboration (Choudhary et al., 2023). As an example, Liang et al. (2020) found that AI applications in healthcare significantly reduce diagnostic risks by enabling the early detection and accurate assessment of severe risks. Similarly, in the financial sector, Xu (2024) found that AI—particularly large language models—effectively mitigates various risks by improving the accuracy of risk assessments, optimizing business decisions, detecting and preventing fraud in real-time, and enhancing customer service. Beyond these technical capabilities, recent studies have revealed important psychological mechanisms underlying AI’s influence on risk-taking willingness. Said et al. (2023) demonstrated that individuals with greater confidence in AI knowledge tend to amplify potential benefits while underestimating risks, which may enhance employees’ willingness to take risks in AI-assisted decision-making contexts. This cognitive bias toward optimistic risk assessment suggests that AI familiarity creates a psychological environment conducive to bolder decision-making. Furthermore, Albashrawi (2025) revealed that generative AI influences users’ risk perception and decision behavior across financial and healthcare contexts through personalized modeling and risk tolerance adaptation. The decision support provided by AI can be viewed as a form of “safety assurance,” encouraging employees to explore more innovative yet potentially riskier solutions (Mariani et al., 2023). The evidence indicates that AI usage provides analytical support while simultaneously reshaping the psychological framework through which employees evaluate and approach uncertain situations, ultimately enhancing their willingness to take risks. Given this, we put forward the following hypothesis:

**H1:**  *There is a positive correlation between AI usage and willingness to take risks*.

### 2.2. The Mediating Role of Self-Efficacy

As a fundamental aspect of social cognitive theory, self-efficacy represents an individual’s belief in their ability to succeed in specific tasks (Bandura, 1997). This research posits that AI usage may enhance employees’ self-efficacy through various mechanisms. First, the information support and decision assistance provided by AI systems may reinforce employees’ perceptions of their abilities. Research by Raisch and Krakowski (2021) indicates that AI effectively reduces employees’ cognitive load by rapidly processing information, analyzing data, and drawing conclusions. When employees become proficient in using AI tools and receive positive feedback, they may attribute this success to an enhancement of their abilities, thereby increasing their self-efficacy. Second, the independence that AI systems offer can alleviate work pressure, thereby enhancing employees’ innovative capacity and self-efficacy (Zheng et al., 2025). Moreover, AI usage increases employees’ creative self-efficacy, which in turn enhances their creativity (Jeong & Jeong, 2025). Finally, AI usage can significantly enhance employees’ technological self-efficacy (Y. Liu et al., 2024). Specifically, AI helps employees to reduce their workload of mechanical and repetitive tasks by simplifying work processes and providing efficient support, thus increasing their confidence and mastery of technology.

Self-efficacy, as a core concept of individual cognition, plays a crucial role in shaping risk-taking behavior. According to SCT, those with a high self-efficacy are more apt to set ambitious goals and to continue striving to accomplish these goals (Bandura, 2001). Employees with a high self-efficacy are inclined to actively learn job-related knowledge and skills, as well as to possess an exploratory spirit and a sense of adventure, which facilitates the cultivation of innovative thinking (Yang et al., 2021). Findings from the work of Lucas et al. (2025) reveal that self-efficacy significantly affects employees’ willingness to take risks, with a higher self-efficacy enhancing the willingness to engage in risk-taking behavior, thereby promoting more proactive entrepreneurial actions. This relationship extends beyond workplace contexts, as demonstrated by D. Liu et al. (2025) in tourism consumption settings, where individual self-efficacy significantly and positively predicts willingness to take risks; when self-efficacy increases due to expanded social roles, consumers become more willing to choose high-risk activities such as skydiving and bungee jumping. In an organizational context, research by Caputo et al. (2025) relating to entrepreneurs indicates that self-efficacy can motivate individuals to take more actions when facing entrepreneurial opportunities, including making riskier investment decisions. Specifically, those with a high self-efficacy often view risks as opportunities rather than potential threats, thereby motivating them to engage in more innovative and risk-taking choices. Similarly, at the leadership level, Kim and Beehr (2023) found that empowering leadership enhances employees’ role-based self-efficacy and perception of meaningful work, which indirectly promotes employees’ risk-taking and entrepreneurial behaviors.

Social cognitive theory emphasizes the idea that environmental factors (such as technological tools) influence behavioral decision-making by affecting individuals’ cognitive evaluations (Bandura, 1986). Therefore, AI usage, as an environmental factor, can enhance employees’ self-efficacy by improving information acquisition capabilities, providing decision support, and reducing cognitive load. Subsequently, this enhanced self-efficacy encourages employees to be more willing to engage in innovative behaviors and risk-related decision-making under uncertainty. Recent empirical studies provide robust support for this mediating mechanism. Q. Zhang et al. (2025) demonstrated that AI usage enhances employee innovative behaviors, with self-efficacy functioning as a crucial mediating factor in this process. Their findings reveal that AI tools enhance employees’ confidence in their capabilities, which subsequently promotes innovative risk-taking behaviors. Furthermore, Yin et al. (2024) examined the dual impact of AI-assistant intelligence on employees’ innovation behavior, revealing that when AI usage enhances self-efficacy, it significantly promotes employees’ willingness to engage in innovative and risk-taking activities. Similarly, research by Liang et al. (2020) indicates that medical AI-assisted diagnostic systems indirectly promote doctors’ willingness to adopt innovative treatment plans by enhancing their professional confidence. Based on this, we put forward the following hypotheses:

**H2:**  *AI usage has a positive impact on self-efficacy*.

**H3:**  *Self-efficacy positively influences willingness to take risks*.

**H4:**  *Self-efficacy acts as a mediator between AI usage and willingness to take risks*.

### 2.3. The Moderating Effect of Learning Goal Orientation

Learning goal orientation is defined as an individual’s drive to enhance their abilities by acquiring new knowledge and skills (Dweck, 1986). Employees who possess a high learning goal orientation generally see challenges as opportunities for learning, seek out feedback actively, and work diligently to acquire new skills (VandeWalle et al., 2001). Conversely, employees with a low learning goal orientation are likely to overlook personal learning and the development of individual skills. Payne et al. (2007) suggest that learning goal orientation is subject to influences from external environments. Consequently, in relation to AI usage, there may be significant disparities among employees with varying goal orientations. Those with a high learning goal orientation often see AI as a valuable resource for enhancing their skill set, believing that AI tools not only improve work efficiency but also enhance their learning journeys. This cognition prompts them to actively use AI to complete complex tasks, thereby enhancing their self-efficacy. They are more likely to experience the “tool integration” effect, perceiving AI capabilities as an extension of their own abilities. The collaboration between employees and AI can boost employees’ confidence when faced with higher work demands, which increases their likelihood of engaging in learning activities and consequently enhances their self-efficacy (A. Chen et al., 2023). Furthermore, those who exhibit a strong learning goal orientation typically have greater confidence in their learning abilities and technology usage (Wang et al., 2025). Consequently, when they achieve success through AI usage, they tend to view this success as a validation of their abilities, which in turn boosts their self-efficacy. Relevant empirical studies also support this view. L. Liu et al. (2024) reported that workers characterized by a high learning goal orientation are inclined to demonstrate robust tool adaptability and confidence when facing challenges and learning new technologies. This indicates that they can swiftly adapt to new tools and technologies, improving their work efficiency. Similarly, research by H. Zhang et al. (2023) reveals that those with high learning goal orientation can fully utilize the resources provided by AI, actively learn, and apply new technologies, thus enhancing their confidence. Therefore, we put forward the following hypothesis:

**H5:**  *Learning goal orientation moderates the relationship between AI usage and self-efficacy, resulting in a more pronounced positive effect of AI usage on self-efficacy for employees who possess a high learning goal orientation than for those with a low orientation*.

### 2.4. The Moderated Mediation Effect

According to social cognitive theory, learning goal orientation, which is a key personal characteristic, may systematically influence how employees perceive and use AI tools, as well as how this usage translates into self-perception and behavioral tendencies. Employees exhibiting a strong learning goal orientation not only integrate AI tools more effectively into their capability systems but may also leverage the resulting self-efficacy to support risk decision-making (BarNir et al., 2011). Specifically, individuals characterized by a high learning goal orientation are inclined to concentrate on developing their competencies and mastering new skills; they may regard AI tools as a resource to enhance their capabilities rather than as external dependencies that replace their own judgment, thereby reducing work-related insecurity (Zhu et al., 2021). Consequently, the self-efficacy gained from technology usage feels more authentic and internalized for them, enabling a more effective translation into a willingness to take risks. Recent empirical evidence supports this perspective, as J. Qian et al. (2025) demonstrated that the higher employees’ learning goal orientation, the more significant the effect of AI usage on promoting innovative behavior through enhancing work absorption, and consequently, employees are more willing to accept challenging and risky tasks. For instance, Ding et al. (2023) indicate that individuals characterized by a high learning goal orientation are better at transforming technological empowerment into actual innovative behavior, with self-efficacy playing a significant mediating role in this process. Similarly, C. Qian and Kee (2023) explored how individual and team-level learning goal orientation influences employee creativity through creative self-efficacy. Conversely, employees who possess a low learning goal orientation do not emphasize learning enhancement and resource acquisition, thus adopting a negative view of AI technology applications, which increases work-related insecurity (H. Zhang et al., 2023). In these situations, even if AI usage may temporarily enhance self-efficacy, this efficacy is likely to stem more from the external tools themselves rather than from an enhancement of intrinsic capabilities, resulting in a weaker impact on the willingness to take risks. From the perspective of SCT, the changing external environment of AI usage influences employees’ learning attitudes and motivations, thereby affecting their willingness to engage in risk-taking behavior to a certain extent (Bandura, 2001). People who possess a high learning goal orientation typically attribute their success under AI assistance to their own learning and capability improvement, thus reinforcing their intrinsic self-efficacy; however, individuals with low learning goal orientations may attribute their success more to external tools, making it difficult to form a lasting sense of self-efficacy. This difference might result in varying effects of self-efficacy on employees’ willingness to take risks based on their levels of learning goal orientation. The following hypothesis is presented in this research:

**H6:**  *Learning goal orientation moderates the mediating effect of self-efficacy on the connection between AI usage and the willingness to take risks; that is, for people who possess high learning goal orientation, AI usage significantly affects willingness to take risks via self-efficacy*.

Table 1 presents the conceptual definitions of variables, empirical support for hypotheses, and theoretical derivation processes in this study, in order to present the theoretical framework in a clear and comprehensible manner. This structured presentation facilitates understanding of how AI usage, self-efficacy, and learning goal orientation influence employees’ willingness to take risks.

**Figure 1:**
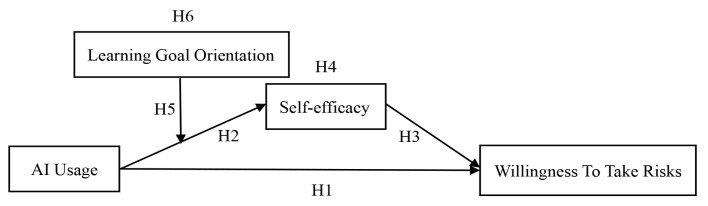
Theoretical model.

Based on established moderated mediation analytical approaches (Zhan et al., 2025; Jeong & Jeong, 2025), our proposed theoretical framework, depicted in Figure 1, positions AI usage as the predictor variable and willingness to take risks as the outcome variable. The model proposes self-efficacy as the underlying mechanism that transmits the influence from AI usage to willingness to take risks. Additionally, learning goal orientation is examined as a moderator that influences this mediation process. This analytical framework follows established practices in organizational behavior research.

## 3. Method

This study employed a quantitative research approach using structured questionnaires to collect data. The research tool consisted of validated scales measuring AI usage, self-efficacy, learning goal orientation, and willingness to take risks. The target sample size of 700 was determined using G*Power 3.1 based on structural equation modeling requirements, considering potential attrition across the multi-wave data collection process.

### 3.1. Procedure and Participants

This study collected data through a multi-wave approach from January 2025 to March 2025. The questionnaires were distributed via the “Credamo” online survey platform, which represents a leading professional data collection service in China with a track record of over 340,000 completed research studies and data quality meeting international academic journal standards. To mitigate potential common method bias from affecting the study results, data were collected in three stages. To reduce the potential influence of consistency motivation and to prevent participants from answering carelessly, the questionnaire included reverse-scoring items. In the first stage, participants were invited to evaluate AI usage and fill out personal demographic details, including gender, age, etc. A total of 700 questionnaires were given out, and 570 of those responses were valid, resulting in a response rate of 81.4%. In the second stage, participants completed the self-efficacy and learning goal orientation scales, which resulted in collecting 426 valid responses. In the third stage, participants were invited to assess their willingness to take risks. By matching questionnaires from the three stages and excluding invalid samples, the final dataset comprised 442 valid samples, achieving a response rate of 63.1%. The 442 valid samples were primarily from Guangdong, Jiangsu, Anhui, Zhejiang, and Shandong provinces, mainly involving industries such as manufacturing (128 participants, accounting for 29%), information technology/internet (126 participants, accounting for 28.5%), and finance and banking (39 participants, accounting for 8.8%). Among the participants, 127 were male (28.7%), and 315 were female (71.3%); the majority (229 participants, or 51.8%) were aged between 31 and 40. Their education levels were relatively high, with 301 participants (68.1%) holding a bachelor’s degree. The selection of industries such as manufacturing, information technology/internet, and finance/banking, for this research is due to the high application of AI technology in these fields, as well as the rich scenarios in intelligent manufacturing, algorithm development, and intelligent risk control, allowing employees to have frequent and in-depth interaction with AI. This provides an ideal research sample to observe how AI usage affects the willingness to take risks. Additionally, the wide distribution of survey samples, balanced gender representation, and reasonable age and educational structure contribute to the overall representativeness of the data sample.

### 3.2. Measures

This research applied well-established scales developed by international scholars. The selected measurement scales for AI usage, learning goal orientation, self-efficacy, and the willingness to take risks have either been widely used in domestic research or have been repeatedly validated for their high reliability and validity. For each measurement scale, a standard “translation–back translation” process was employed, referencing the Chinese versions in the relevant literature and making appropriate modifications based on the local context. All scales used a 5-point Likert scoring method, where participants evaluated the items, with 1 representing “not at all true (disagree)” and 5 representing “completely true (agree).”

AI Usage. The AI usage items were sourced from Tang et al. (2022) and comprise three items, such as “I use artificial intelligence to perform most of my work tasks (α = 0.845).”

Learning Goal Orientation. The items measuring learning goal orientation were based on the measure established by Vandewalle (1997). This scale includes five items, such as “I am willing to seek out challenging work assignments that I can learn a lot from (α = 0.810).”

Self-Efficacy. The scale established by Scholz et al. (2002) was utilized, consisting of 10 items, such as “As long as I try hard, I can always solve difficult problems (α = 0.804).”

Willingness to Take Risks. The scale was adapted from the work of Dewett (2006) and consists of eight items. Example items include “When I think of a good method to improve work, I am willing to try it even if I might fail” and “In order to do my job better, I am willing to take certain risks even if it might lead to failure (α = 0.817).”

Control Variables: As a behavioral tendency among individuals, the willingness to take risks is significantly shaped by demographic factors. In reference to previous studies (Markiewicz & Weber, 2013), this article considers demographic characteristics such as gender, age, education, work experience, position level, and department as potential control variables. The data analysis fully takes into account their possible impacts to enhance the scientific validity and credibility of the research findings. The complete survey questionnaire is presented in Appendix A.

## 4. Results

### 4.1. Statistical Analysis

Statistical analyses of the research data were performed utilizing SPSS 26.0 and Amos 24.0. To begin with, we evaluated common method bias, as well as the discriminant validity of the four variables outlined in the hypothesized model. Subsequently, fundamental descriptive statistical analyses were performed. Finally, hypotheses were tested based on the mediation effect testing procedures in the mediation model, examining the main effects, self-efficacy as a mediator, and learning goal orientation as a moderating variable.

### 4.2. Common Method Bias Test

This research used a multi-wave approach to collect sample data, aiming to control the potential impacts of common method bias and perform a simple test for such bias. First, Harman’s single factor test (Podsakoff et al., 2003) was utilized, which involved conducting a factor analysis on the observed indicators for the four variables—AI usage, self-efficacy, learning goal orientation, and the willingness to take risks. The results demonstrated the extraction of multiple factors, where the first factor accounted for only 32.569% of the variance (less than 40%), which did not exceed the empirical standard. Therefore, the issue of common method bias in the sample data is not significant, allowing progress to the next step of statistical testing.

### 4.3. Confirmatory Factor Analysis

To test the discriminant validity of the four latent variables—AI usage, self-efficacy, learning goal orientation, and the willingness to take risks—a nested structural model was constructed for assessing model fit, for which the findings are presented in Table 2. In every nested model, the fit parameters for the four-factor model met the empirical standards. Moreover, relative to other models, the four-factor approach exhibited the best fit (χ^2^(442) = 678.723; χ^2^/df = 2.365; IFI = 0.908; CFI = 0.894; TLI = 0.907; RMSEA = 0.056). All indices fell within a reasonable range, supporting the rationality of the hypothesized model. Therefore, this study demonstrated a high discriminant validity among the four main variables.

### 4.4. Descriptive Statistics and Correlation Analysis

Table 3 presents the results of the descriptive statistical analysis. AI usage shows a significant positive relationship with self-efficacy (r = 0.560; *p* < 0.01) and with the willingness to take risks (r = 0.531; *p* < 0.01). Self-efficacy also shows a significant positive correlation with the willingness to take risks (r = 0.641; *p* < 0.01). Moreover, a significant positive correlation is found between learning goal orientation and the willingness to take risks (r = 0.553; *p* < 0.01). The descriptive statistical results provide preliminary support for subsequent hypothesis testing, with all variables exhibiting VIF values from 1.011 to 3.042, indicating an absence of multicollinearity issues.

### 4.5. Hypothesis Testing

**Figure 2:**
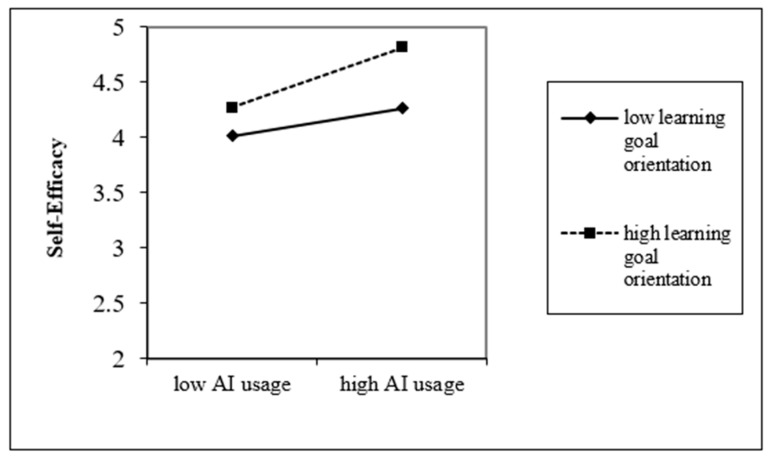
The moderating effect of learning goal orientation on the relationship between AI usage and willingness to take risks.

(1) Main Effects and Mediation Effect Testing. This research employed stepwise regression analysis to test the main effects and the mediation effect of self-efficacy, for which the results are shown in Table 4. Model 6 in Table 4 indicates that AI usage has a significant positive impact on employees’ willingness to take risks (B = 0.493; *p* < 0.001), thereby supporting Hypothesis H1. In Model 2, it is indicated that AI usage significantly influences self-efficacy in a positive manner (B = 0.524; *p* < 0.001). As observed in Model 7, after controlling for the independent variable in the mediation, self-efficacy significantly positively impacts the willingness to take risks (B = 0.487; *p* < 0.001). Therefore, Hypotheses H2 and H3 are also validated. At this point, AI usage is still positively related to the willingness to take risks, but compared to Model 6, the coefficient drops from 0.493 to 0.238, indicating that self-efficacy partially mediates the positive effect of AI usage on the willingness to take risks. To further explore the mediation effect, the Process plugin in SPSS software was utilized, employing the bootstrap method with Model 4 for calculations. Table 5 reports the bootstrap results. The results from 5000 bootstrap resamples indicate that the total effect of AI usage on employees’ willingness to take risks through self-efficacy is 0.236 (*p* < 0.001), which has a 95% confidence interval of [0.196, 0.276], excluding 0. The direct effect is 0.114 (*p* < 0.001) and has a 95% confidence interval of [0.073, 0.155]; the indirect effect stands at 0.122 and has a 95% confidence interval of [0.071, 0.176], which also excludes 0. Therefore, the significant indirect effect further confirms that self-efficacy acts as a mediator between AI usage and the willingness to take risks, validating Hypothesis H4.

(2) Moderating Effect Testing. Model 4 of Table 4 reveals that the interaction term of AI usage and learning goal orientation significantly enhances self-efficacy (B = 0.251; *p* < 0.001), suggesting that learning goal orientation positively influences how AI usage relates to self-efficacy. To better understand the extent and direction of the moderating effect, this study divided the moderating variable into high and low groups based on the mean ± one standard deviation, analyzing how AI usage relates to self-efficacy. A moderating effect diagram is depicted in Figure 2. Compared to employees with a low learning goal orientation, AI usage has a stronger positive impact on the self-efficacy of employees with high learning goal orientation, thus validating Hypothesis H5.

(3) Testing the Moderated Mediation Effect. This study utilized the Process plugin to analyze the moderating impact of learning goal orientation on how AI usage influences employees’ willingness to take risks through self-efficacy, with the results shown in Table 6. When the level of learning goal orientation is high (+1 SD), the estimated value of the mediation effect of AI usage on the willingness to take risks through self-efficacy is 0.112, with a 95% confidence interval of [0.067, 0.163], which does not include 0, indicating that the mediation effect of self-efficacy is significant. Conversely, for low levels of learning goal orientation (−1 SD), the estimated mediation effect is 0.028, with a 95% confidence interval of [−0.014, 0.082], which includes 0, indicating that the mediation effect of self-efficacy is not significant. Additionally, the effect estimate between the high and low groups is 0.084, with a 95% confidence interval of [0.036, 0.110], which does not include 0, demonstrating a significant difference. This indicates that learning goal orientation positively moderates the mediation effect of AI usage on the willingness to take risks through self-efficacy. Therefore, Hypothesis H6 is validated.

## 5. Discussion

### 5.1. Conclusions

Based on social cognitive theory, this research establishes a theoretical model of how AI usage influences employees’ willingness to take risks. The main conclusions are as follows: First, AI usage enhances employees’ willingness to take risks. Second, self-efficacy functions as a partial mediator for the effect of AI usage on risk-taking willingness. Finally, learning goal orientation not only moderates how AI usage relates to self-efficacy but also moderates the mediation of self-efficacy in the connection between AI usage and the willingness to take risks.

First, this study validates that AI usage significantly enhances employees’ willingness to take risks (H1: β = 0.493, *p* < 0.001) and reveals the critical mediating role of self-efficacy in this process (H4: indirect effect = 0.122, 95% CI [0.071, 0.176]). From a mechanistic perspective, AI usage, as an emerging environmental factor, enhances employees’ information acquisition and processing capabilities, reduces their cognitive load, thereby improving decision-making quality and self-efficacy (H2: β = 0.524, *p* < 0.001), and ultimately promoting employees’ willingness to take risks in uncertain environments (H3: β = 0.487, *p* < 0.001). This finding aligns with the core tenet of social cognitive theory, which posits that environmental factors influence individual decision-making behavior through their impact on cognitive abilities (Bandura, 1986). Related empirical studies provide supporting evidence: D. Liu et al. (2025) found in tourism consumption contexts that consumers were more willing to engage in high-risk activities such as skydiving and bungee jumping when their self-efficacy was enhanced through social role expansion. Similarly, Yin et al. (2024) demonstrated that when AI usage enhances employees’ self-efficacy, it significantly promotes their willingness to engage in innovative and risk-taking activities.

Second, learning goal orientation plays a significant positive moderating role between AI usage and willingness to take risks (H5: β = 0.251, *p* < 0.001; H6: difference = 0.084, 95% CI [0.036, 0.110]). Specifically, employees with high learning goal orientation are more inclined to view AI as an effective tool for enhancing their capabilities rather than as an external dependency that replaces personal judgment (Zhu et al., 2021). Meanwhile, individuals with high learning goal orientation typically demonstrate greater confidence in their learning abilities and technology application (Wang et al., 2025), thus enabling them to more effectively transform the efficacy gained from technology usage into willingness to take risks. This perspective also validates the importance of individual characteristics in behavioral choices within social cognitive theory (VandeWalle et al., 2001). Conversely, employees with low learning goal orientation often hold negative attitudes toward AI tools, perceiving them as threats to job security (H. Zhang et al., 2023), thereby weakening their willingness to take risks. This finding is consistent with J. Qian et al. (2025), who demonstrated that the higher employees’ learning goal orientation, the more significant the effect of AI usage in promoting innovative behavior through enhanced work engagement, making employees more willing to undertake challenging and risky tasks.

### 5.2. Theoretical Contributions

This research examines how AI usage influences employees’ willingness to take risks, utilizing social cognitive theory as a framework, thereby expanding the theoretical perspective of current research. Previous research has largely investigated the influence of AI on organizational performance and job design (e.g., Tang et al., 2022; Jia et al., 2024), with relatively few studies examining the mechanisms through which technology influences employees’ willingness to take risks. This research demonstrates that AI usage, as an emerging environmental factor, significantly enhances employees’ willingness to take risks. This finding emphasizes the transformative role of technology application in modern organizations and suggests that managers should consider how technological tools influence employee behavior and psychological responses when advancing digital transformation. From this perspective, this study contributes to the theoretical foundation for assessing employee decision-making behavior regarding technological dependence, supporting the views of related research (Brynjolfsson & McAfee, 2014) and providing a new angle for further investigation into employees’ willingness to take risks.

Furthermore, this study validates the role of self-efficacy as a mediator in how AI usage influences employees’ willingness to take risks, further supporting the core tenet of social cognitive theory regarding the interaction of environmental factors, personal factors, and behavioral decisions. Self-efficacy is enhanced by the application of AI technology in the workplace, as it reduces cognitive load and subsequently increases employees’ willingness to take risks. This mechanism reveals the significant influence of individuals’ internal beliefs on behavioral choices in a rapidly changing technological environment (Bandura, 1997; Y. Liu et al., 2024), further affirming the effectiveness of social cognitive theory in explaining the impact of modern technology on individual behavior.

Finally, the research also finds that learning goal orientation plays a moderating role in the association between AI usage and self-efficacy, illustrating how individual traits affect the efficacy of technology use. Those with a high learning goal orientation are inclined to regard AI as a tool for skill enhancement, making it easier for them to build self-efficacy in the face of uncertainty, further increasing their willingness to take risks (Bandura, 2001). This research not only expands the understanding of how learning goal orientation influences employee behavior but also provides personalized intervention strategies for organizational management practices, helping employees make better use of AI tools.

### 5.3. Management Insights

The findings of this research offer important insights relating to organizational management practices. First, when implementing AI technology, companies should actively cultivate a positive work environment to enhance employees’ self-efficacy. Currently, many employees still experience a degree of anxiety and unease regarding the introduction of AI technology, which can affect their job performance and contribute to job insecurity (W. Chen et al., 2022). Therefore, managers can help employees overcome these psychological barriers by providing the relevant training, technical support, and positive feedback. For example, regular training sessions and workshops can familiarize employees with the use of AI tools and demonstrate, through real-life examples, the support AI can provide in daily work, thereby enhancing employees’ confidence in their abilities. Meanwhile, employees should actively embrace AI technology as a learning opportunity rather than viewing it as a threat, participating proactively in training programs and seeking feedback to develop their technological competencies. Such support not only improves employees’ self-efficacy but also boosts their work efficiency and overall innovative capabilities (Q. Zhang et al., 2025), helping maintain a competitive advantage in a rapidly changing digital environment (Maran et al., 2022).

Secondly, organizations should design personalized training and incentive mechanisms based on employees’ learning goal orientation to encourage proactive learning in conjunction with AI tools. Those with a high learning goal orientation often regard AI as a resource for improving their abilities rather than simply as a substitute (A. Chen et al., 2023). Therefore, companies can develop incentive measures and goal-setting strategies that allow employees to have greater autonomy and a sense of participation. For instance, providing challenging tasks and feedback support to individuals exhibiting high learning goal orientation can promote their active application of AI technology and deep learning. In contrast, for individuals exhibiting a lower learning goal orientation, companies should consider implementing more targeted interventions, such as offering personalized coaching and support, to help them recognize the value of AI and stimulate their willingness to learn. Additionally, employees with lower learning goal orientation should be encouraged to view AI as a capability enhancement tool and actively seek mentorship to develop their technological confidence. Through such differentiated management strategies, organizations can effectively enhance employees’ willingness to take risks, promote overall team innovation capability, and achieve sustainable development in a continuously changing market environment.

## 6. Limitations

This study has the following limitations: First, in terms of sample collection, to control for common method bias, we conducted data collection at multiple time points, gathering data on different research variables across three stages. While this partially addresses the temporal issues in the causal relationships of the theoretical model, it still does not completely eliminate the presence of common method variance. Therefore, future research could employ experimental methods to collect data, ensuring that the results are more precise. Second, regarding moderating variables, this study focused on the individual traits of employees. Future research could consider incorporating other contextual variables, such as team atmosphere and leadership style, in order to gain a more comprehensive understanding of how AI usage affects employees’ psychology and behavior. Third, this study’s sample is largely drawn from specific industries, which could restrict the generalizability of the findings. Since culture, work environments, and levels of technological dependence vary across different industries, future research could expand into different fields in order to validate the generalizability and applicability of the findings. This would contribute to a more comprehensive understanding of the impact of AI usage on employees’ psychology and behavior.

## Figures and Tables

**Table 1:** Key Variables and Theoretical Support.

Variable	Conception	Hypotheses and Empirical Support	Theoretical Support
AI usage	Employees engaging with various forms of AI to perform relevant tasks, including analysis, computation, and decision-making (Tang et al., 2022).	H1 (Xu, 2024; Said et al., 2023; Albashrawi, 2025; Mariani et al., 2023)	Based on social cognitive theory, AI usage as an external environmental change reduces employees’ cognitive burden and increases their confidence in handling work and tasks. In turn, this enhanced self-efficacy improves willingness to take risks. Learning goal orientation, as an individual characteristic, moderates the extent to which AI usage influences self-efficacy and subsequently affects the degree of willingness to take risks. Thus, SCT provides a theoretical framework for linking the relationships among AI usage, self-efficacy, learning goal orientation, and willingness to take risks.
		H2 (Zheng et al., 2025; Jeong & Jeong, 2025; Y. Liu et al., 2024)	
Willingness to take risks	The tendency to accept certain job-related risks for the sake of achieving positive outcomes at work (Dewett, 2006).	H3 (Lucas et al., 2025; D. Liu et al., 2025; Kim & Beehr, 2023)	
		H4 (Q. Zhang et al., 2025; Yin et al., 2024; Liang et al., 2020)	
Self-efficacy	An individual’s belief in their ability to succeed in specific tasks (Bandura, 1997).	H5 (Wang et al., 2025; L. Liu et al., 2024; H. Zhang et al., 2023)	
Learning goal orientation	An individual’s drive to enhance their abilities by acquiring new knowledge and skills (Dweck, 1986).	H6 (J. Qian et al., 2025; Ding et al., 2023; C. Qian & Kee, 2023)	

**Table 2:** Results of confirmatory factor analysis.

Model	χ^2^	df	χ^2^/df	IFI	CFI	TLI	RMSEA
1. Four-factor model (AIU, LGO, SE, WTR)	678.723	287	2.365	0.908	0.894	0.907	0.056
2. Three-factor model (AIU + LGO, SE, WTR)	1127.894	296	3.81	0.803	0.783	0.802	0.080
3. Three-factor model (AIU + SE, LGO, WTR)	1160.794	296	3.922	0.796	0.794	0.774	0.081
4. Three-factor model (AIU + WTR, LGO, SE)	1196.331	296	4.04	0.787	0.786	0.765	0.083
5. Two-factor model (AIU + WTRLGO + SE)	1377.565	298	4.623	0.745	0.743	0.72	0.091
6. Single-factor model (AIU + SE + LGO + WTR)	1464.489	299	4.898	0.724	0.723	0.698	0.094

Note: N = 442; AIU = AI usage; LGO = learning goal orientation; SE = self-efficacy; WTR = willingness to take risks. The “+” sign indicates combined variables.

**Table 3:** Results of descriptive statistical analysis (N = 442).

	M	SD	1	2	3	4	5	6	7	8	9	10
1. Gender	1.713	0.453										
2. Age	2.658	0.669	0.079									
3. Edu	2.206	0.576	0.027	0.124 **								
4. Work	1.993	0.977	0.042	0.805 **	−0.038							
5. Position	2.029	0.958	0.082	0.486 **	0.240 **	0.444 **						
6. Section	3.380	2.168	0.005	0.054	−0.155 **	0.156 **	−0.024					
7. AIU	3.628	0.910	−0.013	0.021	0.121 *	−0.059	0.072	−0.318 **	**(0.845)**			
8. LGO	4.259	0.519	−0.028	0.044	0.07	0.008	0.185 **	−0.196 **	0.579 **	**(0.810)**		
9. SE	4.217	0.376	−0.101 *	0.055	0.197 **	−0.005	0.174 **	−0.231 **	0.560 **	0.572 **	**(0.804)**	
10. WTR	4.221	0.435	−0.085	−0.042	0.145 **	−0.063	0.101 *	−0.258 **	0.531 **	0.553 **	0.641 **	**(0.817)**

Note: ** = *p* < 0.01; * = *p* < 0.05; AIU = AI usage; LGO = learning goal orientation; SE = self-efficacy; WTR = willingness to take risks. The bold values represent Cronbach’s α coefficient.

**Table 4:** Results of multiple linear regression analysis (N = 442).

Variable		SE				WTR		
		Model 1	Model 2	Model 3	Model 4	Model 5	Model 6	Model 7
Control Variable	Gender	−0.12 *	−0.106 **	−0.095 **	−0.083 *	−0.089	−0.079 *	−0.028
	Age	0.027	−0.023	−0.004	−0.004 *	−0.098	−0.145 *	−0.133 *
	Edu	0.125 *	0.102 *	0.114 **	0.102 **	0.093	0.071	0.021
	Work	−0.06	0.003	0	−0.013	0.004	0.06	0.058
	Position	0.16 **	0.129 **	0.066	0.056	0.126 *	0.096 *	0.034
	Section	−0.2 ***	−0.044	−0.039	−0.023	−0.235	−0.089	−0.067
Independent Variable	AIU		0.524 ***	0.323 ***	0.3 ***		0.493 ***	0.238 ***
Moderating Variable	LGO			0.355 ***	0.557 ***			
Interaction Term	AIU*LGO				0.251 ***			
Intermediary Variable	SE							0.487 ***
	R^2^	0.112	0.356	0.436	0.464	0.098	0.313	0.466
	ΔR^2^	0.112	0.243	0.324	0.351	0.098	0.215	0.368
	F	9.173 ***	34.199 ***	41.834 ***	41.512 ***	7.889 ***	28.302 ***	47.243 ***

Note: N = 442; * *p* < 0.05; ** *p* < 0.01; *** *p* < 0.001. AIU = AI usage; LGO = learning goal orientation; SE = self-efficacy; WTR = willingness to take risks.

**Table 5:** Mediator effect test results.

Paths: AI Usage → Self-Efficacy → Willingness to Take Risks				
Model	Efficiency Value	Standard Error	95% Confidence Interval	
Total Effect	0.236	0.020	0.196	0.276
Direct Effect	0.114	0.021	0.073	0.155
Indirect Effect	0.122	0.027	0.071	0.176

**Table 6:** Moderated mediating effect test results.

Independent Variable	Dependent Variable	Moderating Variable Grouping	Mediating Effect Estimate	Standard Error	95% Confidence Interval	
AI Usage	Willingness to Take Risks	eff1 (M − 1 SD)	0.028	0.024	−0.014	0.082
		eff2 (M)	0.084	0.019	0.036	0.110
		eff3 (M + 1 SD)	0.112	0.025	0.067	0.163

## Data Availability

The data collected and analyzed in this study can be provided upon a reasonable request.

## Appendix A. Survey Questionnaire

Dear Sir/Madam:
Hello!

Thank you very much for taking your valuable time to participate in this survey. First, we solemnly promise that the survey data will only be used for academic research and will never disclose any personal information to third parties. Please feel free to answer with confidence. There are no right or wrong answers; you only need to make choices based on your actual feelings and thoughts. Please do not have any concerns. Your participation is the key to the success of this research. Thank you again for your enthusiastic help! We wish you success in your work and a happy life!

Q1. Please select your gender [Single Choice]
  - Male
  - Female
Q2. Please select your age group [Single Choice]
  - 0–20 years old
  - 21–30 years old
  - 31–40 years old
  - 41–50 years old
  - Over 51 years old
Q3. Please select your highest education level [Single Choice]
  - Associate degree or below
  - Bachelor’s degree
  - Master’s degree
  - Doctoral degree
Q4. Please select your years of work experience [Single Choice]
  - Less than 5 years
  - 6–10 years
  - 11–15 years
  - 16–20 years
  - More than 21 years
Q5. Your current position level is: [Single Choice]
  - General employee (no management responsibilities)
  - Frontline manager (supervisor/team leader level)
  - Middle manager (department manager level)
  - Senior manager (director level and above)
Q6. Your industry type is: [Single Choice]
  - Information Technology/Internet/Software
  - Finance/Banking/Insurance
  - Manufacturing/Industry
  - Healthcare/Pharmaceutical/Health
  - Real Estate/Construction
  - Professional Services (Consulting/Legal/Accounting, etc.)
  - Other
Q7. The following questions are about your specific use of artificial intelligence (AI) in actual work. Artificial intelligence here includes but is not limited to: ChatGPT, intelligent assistants, intelligent analysis tools, automation systems, etc. Please make selections based on your actual usage.
Scale: 1 = Strongly Disagree, 2 = Disagree, 3 = Neutral, 4 = Agree, 5 = Strongly Agree
  1. I used artificial intelligence to carry out most of my job functions.
  2. I spent most of the time working with artificial intelligence.
  3. I worked with artificial intelligence in making major work decisions.
Q8. The following questions are about your attitude toward learning and development at work. Please make selections based on your true thoughts and feelings.
Scale: 1 = Strongly Disagree, 2 = Disagree, 3 = Neutral, 4 = Agree, 5 = Strongly Agree
  1. I am willing to seek out challenging work assignments that I can learn a lot from.
  2. I often look for opportunities to develop new skills and knowledge.
  3. I enjoy challenging and difficult tasks at work where I’ll learn new skills.
  4. For me, development of my work ability is important enough to take risks.
  5. I prefer to work in situations that require a high level of ability and talent.
Q9. The following items describe an individual’s attitudes and abilities when facing various situations. Please evaluate based on your true feelings.
Scale: 1 = Strongly Disagree, 2 = Disagree, 3 = Uncertain, 4 = Agree, 5 = Strongly Agree
  1. I can always manage to solve difficult problems if I try hard enough.
  2. If someone opposes me, I can find the means and ways to get what I want.
  3. I am certain that I can accomplish my goals.
  4. I am confident that I could deal efficiently with unexpected events.
  5. Thanks to my resourcefulness, I can handle unforeseen situations.
  6. I can solve most problems if I invest the necessary effort.
  7. I can remain calm when facing difficulties because I can rely on my coping abilities.
  8. When I am confronted with a problem, I can find several solutions.
  9. If I am in trouble, I can think of a good solution.
  10. I can handle whatever comes my way.
Q10. The following items describe your attitude toward risk and innovation at work. Please evaluate based on your true thoughts.
  Scale: 1 = Strongly Disagree, 2 = Disagree, 3 = Uncertain, 4 = Agree, 5 = Strongly Agree
  1. When I think of a good way to improve the way I accomplish my work, I will risk potential failure to try it out.
  2. I will take a risk and try something new if I have an idea that might improve my work, regardless of how I might be evaluated.
  3. I will take informed risks at work in order to get the best results, even though my efforts might fail.
  4. I am willing to go out on a limb at work and risk failure when I have a good idea that could help me become more successful.
  5. I don’t think twice about taking calculated risks in my job if I think they will make me more productive, regardless of whether or not my efforts will be successful.
  6. Even if failure is a possibility, I will take informed risks on the job if I think they will help me reach my goals.
  7. When I think of a way to increase the quality of my work, I will take a risk and pursue the idea even though it might not pan out.
  8. In an effort to improve my performance, I am willing to take calculated risks with my work, even if they may not prove successful.
